# Risk Factors for Pre-eclampsia and Eclampsia Disorders in Tertiary Care Center in Sukkur, Pakistan

**DOI:** 10.7759/cureus.6115

**Published:** 2019-11-10

**Authors:** Shoaibunnisa Soomro, Raj Kumar, Hazooran Lakhan, Faizan Shaukat

**Affiliations:** 1 Obstetrics and Gynecology, Ghulam Muhammad Mahar Medical College and Hospital, Sukkur, PAK; 2 Cardiology, Ghulam Muhammad Mahar Medical College and Hospital, Sukkur, PAK; 3 Internal Medicine, Jinnah Postgraduate Medical Centre, Karachi, PAK

**Keywords:** preeclampsia, eclampsia, hypertensive disorder of pregnancy, pakistan

## Abstract

Introduction

Pakistan has the third-highest incidence of maternal, fetal, and child mortality, according to a recent systemic analysis of global mortality. Thirty-four percent of maternal deaths are attributed to eclampsia among those who are admitted into tertiary care hospitals in Pakistan for delivery. In this study, we determine the risk factors associated with pre-eclampsia and eclampsia in the rural city of Sukkur, Pakistan.

Methods and materials

In this prospective observational study, a semi-structured questionnaire was developed to record information about maternal age, education status, parity, the status of chronic hypertension, gestational diabetes, anemia, body mass index (BMI), and history of cardiac disease of all women attending the antenatal clinic. Women diagnosed with pre-eclampsia and eclampsia were placed in a group, and their characteristics were compared with women with any pre-eclampsia and eclampsia.

Results

The incidence of pre-eclampsia and eclampsia was 5.6% (n=112/2212). Identified risk factors for pre-eclampsia and eclampsia included hypertension (28.7%), gestational diabetes (25.9%), anemia (14.9%), maternal age > 35 years (9.3%), BMI greater than 30 kg/m^2^ (8.1%) and 35 kg/m^2^ (11.7%), nulliparity (6.5%), unbooked status (i.e., lack of antenatal care; 6.4%), and low education level (5.8%).

Conclusion

It is important to identify the markers for pre-eclampsia and eclampsia, as they will help physicians and caregivers to reduce maternal and fetal mortality and the complications associated with it.

## Introduction

Pre-eclampsia and eclampsia have a broad influence and impact, not only on maternal health but also on perinatal health, particularly in the developing world. They are responsible for almost a third of a million deaths in low- and middle-income settings and account for more than six million perinatal deaths [1]. Pakistan is the sixth most populated country and has the third-highest burden of maternal, fetal, and child mortality, according to a recent systemic analysis of global mortality [2]. Thirty-four percent of maternal deaths are attributed to eclampsia among those who are admitted into tertiary care hospitals in Pakistan [3].

The highest risk factors for pre-eclampsia in low- and middle-income countries - based on a secondary analysis of the World Health Organization global survey on maternal and perinatal health - are socio-demographic variables such as maternal age > 30 years and low education. Other than socio-demographic variables, clinical factors such as chronic hypertension, obesity, and severe anemia are also responsible for pre-eclampsia and eclampsia [1]. Another study revealed additional risk factors for pre-eclampsia, including a history of pre-eclampsia, multiple gestations, a pre-pregnancy BMI of greater than 24 kg/m2, age > 34 years, nulliparity, urinary tract infection, or working during pregnancy [4]. The objective of this study is to assess the risk factors and identify them as precursors to pre-eclampsia and eclampsia. The results of this study may help clinicians provide extra attention and surveillance to women at risk of pre-eclampsia and eclampsia to reduce maternal complications and fetal mortality.

## Materials and methods

A prospective observational study was conducted in the Obstetrics and Gynaecology Department of Ghulam Muhammad Mahar Medical College and Hospital from January 1 to December 31, 2018. The study was approved by the institutional review board, and informed consent was obtained from all patients. All patients who delivered during the study period were included.

A semi-structured questionnaire was completed, which included information about maternal age, education status, parity, status of chronic hypertension, gestational diabetes, anemia, body mass index, and history of cardiac disease. All questionnaires were completed at the time of the antenatal visit in the third trimester. Women who were visiting for the first time (i.e., those with no previous antenatal checkup) at 32 weeks or more were considered unbooked in this study. Pre-eclampsia was labeled where there was an onset at more than 20 weeks’ gestational age of 24-hour proteinuria ≥30 mg/day and a systolic blood pressure >140 mmHg or diastolic blood pressure ≥90 mmHg as measured twice, using an appropriate cuff, four to six hours and less than seven days apart. Pre-eclampsia with seizures was considered as eclampsia [5]. Exclusion criteria included patients who did not consent to participate or those who were received in such an emergency that they were in delirium and were unable to communicate. Data were analyzed using IBM SPSS Statistics for Windows, Version 22.0. (IBM Corp., Armonk, NY). Mean was calculated for numerical values such as age and blood pressure at time of admission. Frequencies were calculated for risk factors.

## Results

There were 2212 deliveries conducted in the hospital during the study period, of which 2012 deliveries were included in the study. Of those included in the study, there were 112 (5.56%) cases of pre-eclampsia and eclampsia. The mean age of the participants was 23 ± 5 years. The mean systolic pressure was 160.95 ± 13.86 mmHg. The mean diastolic pressure was 103.68 ± 6.291 mmHg. The mean gestational age was 35.9 ± 2.84 weeks. The risk factors for pre-eclampsia and eclampsia are given in Table 1.

**Table 1:** Risk factors for pre-eclampsia and eclampsia

Characteristics	Total no. of patients (n=2012)	Patients with eclampsia/pre-eclampsia (n=112)
Maternal age (years)		
<20	418 (20.7%)	13 (3.1%)
20 to 29	981 (48.7%)	49 (4.9%)
30 to 34	301 (14.9%)	21 (6.9%)
≥35	312 (15.5%)	29 (9.3%)
Education		
None	1214 (60.3%)	71 (5.8%)
Primary	501 (24.9%)	28 (5.5%)
Secondary	246 (12.2%)	11 (4.5%)
Tertiary	51 (2.5%)	2 (3.9%)
Body Mass Index (BMI kg/m^2^)		
<20	517 (25.6%)	13 (2.5%)
20 to <26	714 (35.4%)	23 (3.2%)
26 to <35	421 (20.9%)	34 (8.1%)
≥35	360 (17.8%)	42 (11.6%)
Parity		
Nulliparous	917 (45.5%)	61 (6.5%)
Multiparous	1095 (54.4%)	51 (4.6%)
Chronic hypertension		
Yes	21 (1.0%)	6 (28.7%)
No	1991 (98.9%)	106 (5.3%)
Gestational diabetes		
Yes	27 (1.3%)	7 (25.9%)
No	1985 (98.6%)	105 (5.2%)
Cardiac disease		
Yes	9 (0.4%)	2 (22.2%)
No	2003 (99. 5%)	110 (5.4%)
Severe anemia		
Yes	161 (8.0%)	24 (14.9%)
No	1851 (92.0%)	88 (4.7%)
Booking status		
Booked	701 (32.8%)	38 (5.4%)
Unbooked	1311 (65.2%)	84 (6.4%)

## Discussion

Pakistan is third on the list of countries with the highest incidence of maternal, fetal, and child mortality [1]. According to a study published by Shah et al., almost one-third of maternal deaths were due to a hypertensive disorder of pregnancy [3]. An Indian study stated that placental abruption and postpartum hemorrhage were the most common complications in pregnant women with pre-eclampsia and eclampsia. Other complications experienced were disseminated intravascular coagulation, acute renal failure, acute respiratory distress syndrome, posterior reversible encephalopathy syndrome, and pulmonary edema [5].

In our study, the prevalence of pre-eclampsia and eclampsia was approximately 5%. This is comparable considering the prevalence of pre-eclampsia in developing countries ranges from as low as 1.8% to as high as 16.7% [6]. In our study, maternal age, low level of education, nulliparity, chronic hypertension, cardiac disease, gestational diabetes, obesity, severe anemia, and unbooked status were associated with increased risk of eclampsia and pre-eclampsia.

In our study, women ages 35 years and older had a higher prevalence (9.29%) of pre-eclampsia and eclampsia. This result was consistent with the findings in Bilano et al., which also stated that increasing maternal age is a predisposing factor in pre-eclampsia and eclampsia [1]. Lamminpää et al., in their work, found that women of advanced age were 1.5 times more likely to develop eclampsia as compared to women younger than 35 years [7]. The exact mechanism of advanced maternal age contributing to eclampsia is not properly understood, but it may be related to aging of uterine vessels [8].

In our study, as the BMI increased, the prevalence of pre-eclampsia and eclampsia increased. Sohlberg et al., in their work, stated that high BMI increases the risk of pre-eclampsia of all severity [9]. While the exact mechanism might not be clear, it may be related to increased oxidative stress, inflammation, and altered vascular function [10].

In our study, pregnant women with chronic hypertension had a far higher incidence of eclampsia and pre-eclampsia as compared to those without chronic hypertension (28.57% vs. 5.32%). The result was consistent with findings in Bilano et al., which also stated that pregnant women with chronic hypertension were strongly linked with the development of eclampsia and pre-eclampsia [1]. Chronic hypertension is a risk factor for eclampsia, and, in turn, eclampsia is associated with high blood pressure later in life. Therefore, the United States Preventive Services Task Force recommends measuring blood pressure throughout pregnancy to screen for pre-eclampsia [11].

In our study, pre-eclampsia and eclampsia were higher in pregnant women, at term, who were unbooked and did not attend antenatal care. In these cases, lack of antenatal care resulted in missing out on milder signs of pre-eclampsia during early pregnancy. Adequate antenatal care plays an essential role in the management of pre-eclampsia and the prevention of eclampsia [1]. Our study results also implied that lower educational status is also a risk factor for the development of pre-eclampsia and eclampsia. The possible explanation is that usually a low education level is associated with low socioeconomic status and improper antenatal care, and lack of awareness may have led to the development of pre-eclampsia [1].

While our study added to the limited data available regarding risk factors of pre-eclampsia and eclampsia in rural areas of Pakistan, it had a few limitations as well. First, since it was a single-institute study, it cannot be generalized to a broader population. Since the data were collected during pregnancy, we could not determine maternal and fetal outcomes. It is important to create awareness about these risk factors to reduce the mortality rate associated with eclampsia and pre-eclampsia. Flyers and brochures in local languages should be distributed to raise awareness about risk factors. Local health works should be involved in door-to-door activity. Blood pressure should be monitored regularly during pregnancy, and efforts should be made to reduce weight before conceiving.

## Conclusions

In our study, maternal age, low level of education, nulliparity, chronic hypertension, cardiac disease, gestational diabetes, obesity, severe anemia, and unbooked status were associated with an increased risk of eclampsia and pre-eclampsia. It is important to aware woman of child bearing age of all the possible risk factors for eclampsia and pre-eclampsia and focused attention should be paid to those at risk of developing eclampsia and preeclampsia to reduce maternal and fetal mortality. 
